# Female sex hormones exacerbate retinal neurodegeneration

**DOI:** 10.1126/sciadv.adr6211

**Published:** 2025-04-11

**Authors:** Ashley A. Rowe, Mauricio J. Velasquez, Jacob W. Aumeier, Sofia Reyes, Tiffany Yee, Emily R. Nettesheim, Jeffrey G. McDonald, Katherine J. Wert

**Affiliations:** ^1^ Department of Ophthalmology, UT Southwestern Medical Center, Dallas, TX, USA.; ^2^ Center for Human Nutrition, UT Southwestern Medical Center, Dallas, TX, USA.; ^3^ Department of Molecular Genetics, UT Southwestern Medical Center, Dallas, TX, USA.; ^4^ Department of Molecular Biology, UT Southwestern Medical Center, Dallas, TX, USA.; ^5^ Peter O’Donnell, Jr. Brain Institute, UT Southwestern Medical Center, Dallas, TX, USA.; ^6^ Hamon Center for Regenerative Science and Medicine, UT Southwestern Medical Center, Dallas, TX, USA.

## Abstract

Neurodegenerative disorders such as Alzheimer’s disease and macular degeneration represent major sources of human suffering, yet factors influencing disease severity remain poorly understood. Sex has been implicated as one modifying factor. Here, we show that female sex is a risk factor for worsened outcomes in a model of retinal degeneration and that this susceptibility is caused by the presence of female-specific sex hormones. The adverse effect of female sex hormones was specific to diseased retinal neurons, and depletion of these hormones ameliorated this phenotypic effect, while reintroduction worsened rates of disease in females. Transcriptional analysis of retinas showed significant differences between genes involved in pyroptosis, inflammatory responses, and endoplasmic reticulum stress–induced apoptosis between males and females with retinal degeneration. These findings provide crucial insights into the pathogenesis of neurodegenerative diseases and how sex hormones can affect disease severity. These findings have far-reaching implications for clinical trial design and the use of hormonal therapy in females with certain neurodegenerative disorders.

## Teaser

Circulating female sex hormones worsen vision loss in a model for a common inherited retinal degenerative disease.

## INTRODUCTION

Neurodegenerative disorders represent major sources of human suffering, yet the factors influencing disease severity remain poorly understood. Sex has been implicated as one such factor in the pathogenesis of neurodegenerative diseases, such as Alzheimer’s disease, Parkinson’s disease, and age-related macular degeneration (*1*–*5*). Sex differences in disease severity can be the result of many modifiers, including differences in anatomy, sex hormone concentrations, differentially expressed genes, and epigenetic differences between males and females (*6*). In several forms of neurodegeneration, it has been shown that women experience worsened outcomes compared to men, but the reasons for this remain poorly understood. Thus, there is an urgent need to close the gap in our understanding of these sex-related differences to provide better care and treatment for women with these conditions.

Retinitis pigmentosa (RP) is a retinal neurodegenerative disease in which the photoreceptors, the primary light-sensing neurons responsible for vision, undergo a progressive and irreversible degeneration and eventual death. As these important retinal cells die, patients experience vision loss that initiates as night blindness and progresses to full blindness. One major obstacle in finding treatments for patients is the high genetic heterogeneity that exists in RP. There are more than 75 genes that are known to cause an RP phenotype, spanning autosomal recessive, dominant, and X-linked forms (*7*, *8*). While the hope is that molecular pathways are shared across the multitude of genetic forms of RP, understanding factors affecting unique forms of RP is also important for precision medicine approaches to treating patients with this neurodegenerative disease. Now, retinal degenerative diseases, such as RP, have no treatment options available. Therefore, patient follow-up by clinicians is not always performed at set time windows during disease progression, complicating investigation into potential sex differences in disease. As there are no treatment options, many clinicians do not perform sequencing to determine the causal gene and mutation for each patient. In addition, the genetic heterogeneity, variable age of onset, and different testing parameters between clinics further confound research investigation. This emphasizes the importance of uncovering how sex and hormone concentrations play a role in neurodegenerative disease progression, to better inform clinical care.

Sex hormones carry out biological functions in various cells of the body by binding to hormone receptors either on the cell surface or intracellularly (*9*–*18*). mRNA and protein for these receptors are present in the retinal tissue (*13*), indicating that they play a role within the retina, but their exact function remains unknown. However, recent studies have noted sex differences in mouse models for retinal degenerative disease. For instance, one study published that the *rd10* mouse, carrying a mutation in the rod cyclic guanosine 3′,5′-monophosphate (cGMP)–specific 3′,5′-cyclic phosphodiesterase 6 catalytic subunit β that acts to hydrolyze cGMP in the rod photoreceptors to allow for cGMP-gated channel closure for phototransduction (*19*, *20*), caused retinas from female mice to degenerate on a faster timeline (*21*). Another study has noted that a *Herc3^−/−^* mouse model with retinal thinning and subretinal microglia, caused by loss of an E3 ubiquitin ligase, showed a more rapid rate of retinal thinning and accumulation of fundus spots in females compared to males (*22*). Furthermore, a study investigated publicly available human and mouse retina transcriptomic datasets and confirmed sex differences in the retina during aging and various retinal degenerative diseases, including RP (*23*). Sex has also been shown to affect metabolic activity within the brain and retinal tissues and, therefore, can likely alter disease susceptibility between males and females (*24*). Although multiple models of retinal degenerative disease show worsened outcomes for females, indicating a role for sex as a modifier of a large cohort of retinal neurodegenerative conditions, studies have not yet linked these sex differences to sex hormones or their mechanistic actions within the retina.

In this study, we used the well-documented mouse model of autosomal dominant RP (*25*–*32*), the most common form of this neurodegenerative disease (*33*), caused by a proline-to-histidine substitution at position 23 of rhodopsin (RHO P23H) (*34*) to investigate whether sex differences affect the retinal degenerative phenotype. We found that female sex was associated with more rapid neurodegeneration and that this effect was caused by female-specific circulating sex hormones using both hormone depletion and hormone reintroduction methods. We further assessed the effects of hormone depletion and reintroduction by developing a method that allowed for the detection of sex hormone concentrations within the neural retina using wild-type, hormone-depleted, and hormone-addition conditions. Investigation into cell death pathways showed significant changes in particular pathways—such as apoptosis—during RP disease, with RP female mice displaying significant changes compared to RP males in two critical genes related to pyroptosis, inflammatory, and endoplasmic reticulum stress–induced apoptotic responses.

Current knowledge remains limited on sex hormones and whether they play a role in the mammalian retina, in either healthy or diseased states. As sex hormones are among the most widely prescribed medications for women in the United States (*35*, *36*), there is a critical need to understand how such a widely prescribed medication affects chronic conditions and reevaluate the safety of these medications for patients with certain forms of RP and other neurodegenerative disorders. Our data highlight an important systemic hormone-dependent interaction of the female sex hormones with the pathology and progression of an inherited retinal dystrophy that was previously thought to be unaffected by biological sex.

## RESULTS

### Female sex is associated with severity of retinal neurodegeneration

**Fig. 1.**
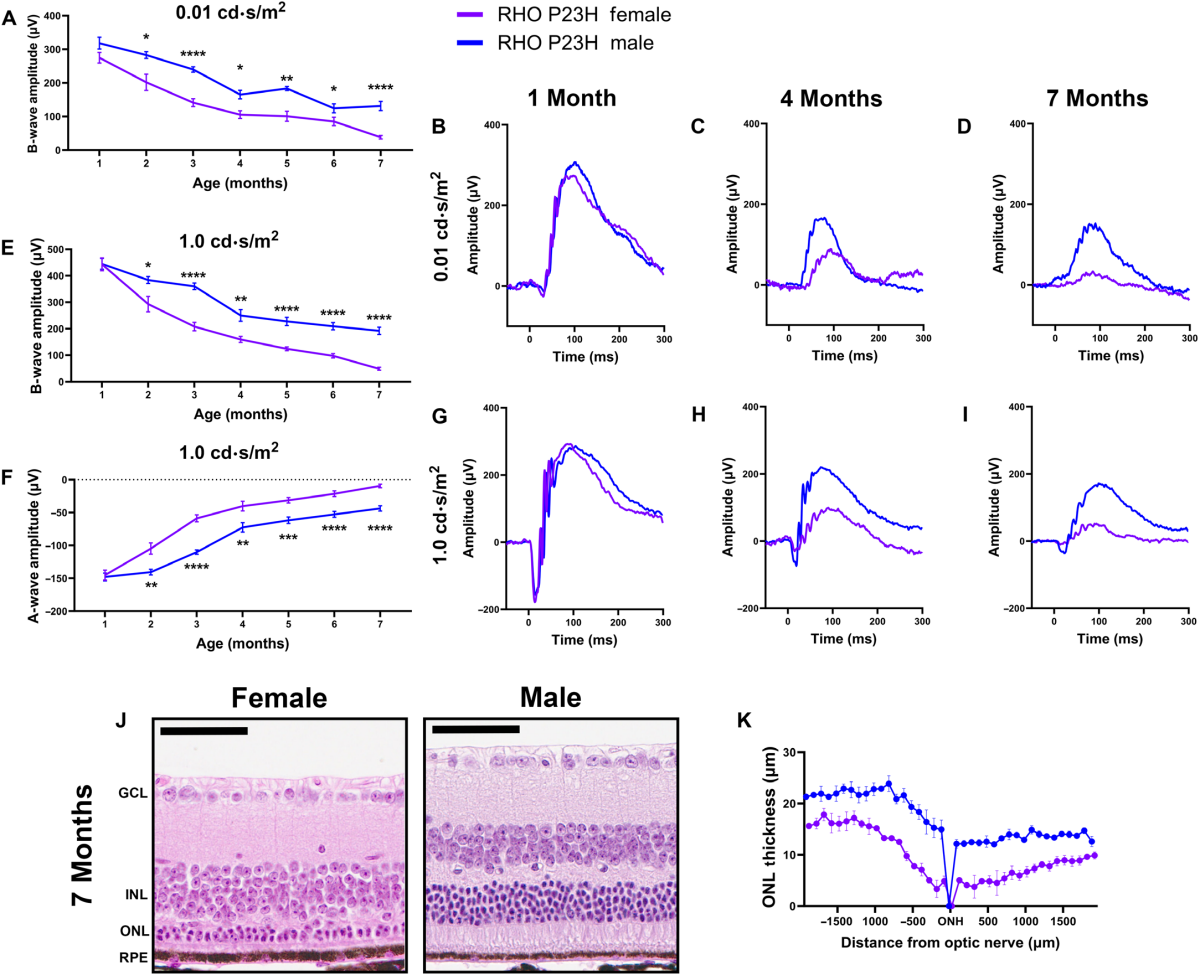
RHO P23H mice display sexually dimorphic loss of photoreceptor function and survival. (**A**) Scotopic ERG (0.01 cd·s/m^2^) b-wave amplitude values for male (blue) and female (purple) RHO P23H mice from 1 to 7 months of age. (**B** to **D**) Representative 0.01 cd⋅s/m^2^ scotopic ERG traces at 1, 4, and 7 months of age. (**E**) Scotopic ERG (1.0 cd·s/m^2^) b- and (**F**) a-wave amplitudes from 1 to 7 months of age. (**G** to **I**) Representative 1.0 cd·s/m^2^ scotopic ERG traces at 1, 4, and 7 months of age. Error bars = SEM. *P < 0.05, **P < 0.01, ***P < 0.001, and ****P < 0.0001. Statistical analysis performed by multiple unpaired *t* test with a Holm-Šídák correction with α = 0.05. *N* ≥ 12 eyes per group. (**J**) Representative hematoxylin and eosin–stained histology from 7-month-old RHO P23H female and male mouse retinas. GCL, ganglion cell layer; INL, inner nuclear layer; RPE, retinal pigmented epithelium. Scale bars, 50 μm. (**K**) Quantification of ONL thickness measured every 100 μm on either side of the optic nerve head (ONH). Statistics for ONL quantification are available in data S1. *N* ≥ 3 mice per group with 38 measurements per retina.

We performed electroretinography (ERG) to test the visual function of RHO P23H female and male mice. Mice underwent scotopic ERG every month through 7 months of age. While both male and female mice had continued neurodegeneration for more than 7 months, consistent with the RP phenotype, female mice displayed a significantly faster decline in visual function beginning at 2 months of age. As rod photoreceptors sense single photons of light, a dark-adapted, dim light intensity was used to isolate the b-wave amplitude response indicative of rod-specific function. These 0.01 cd·s/m^2^ scotopic recordings were significantly lower in female mice compared to male littermates through 7 months of age (Fig. 1A). Scotopic recordings (1.0 cd·s/m^2^), a brighter light intensity that reflects visual function from the photoreceptors through to the bipolar cells, followed a similar outcome, where female mice had a significantly lower visual response in both the b-wave (bipolar cell function; Fig. 1E) and a-wave (rod and cone photoreceptor function) amplitudes (Fig. 1F). Representative ERG traces show the loss of retinal function in female RHO P23H mice compared to males (Fig. 1, B to D and G to I). We next analyzed the structural changes in the retina to assess whether the sexually dimorphic nature was solely a functional loss in the photoreceptor neurons or whether it reflected a difference in cell survival. We found that female RHO P23H mice had a thinner outer nuclear layer (ONL), comprising the photoreceptor nuclei, than male counterparts at all 38 regions of the retina measured—19 measurements spanning 100-μm distances from each side of the optic nerve head—at 7 months of age (Fig. 1, J and K).

### Removal of gonadal sex hormones depletes hormone levels within the mammalian retina

**Fig. 2.**
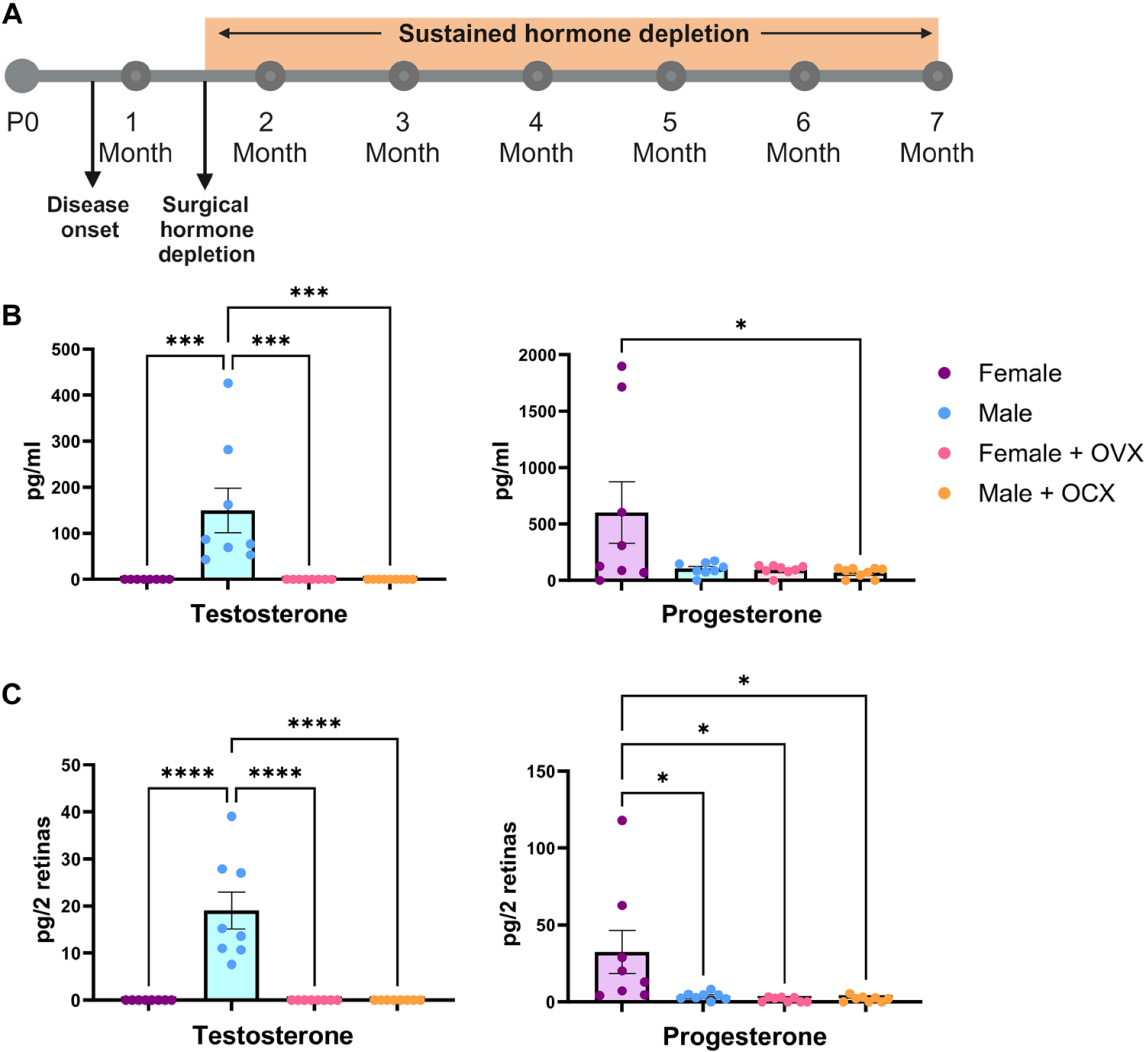
Surgical gonadectomy depletes sex hormones in the serum and retina. (**A**) Timeline depicting the surgical procedure at 6 weeks of age, after disease onset, followed by sustained hormone depletion through the duration of the study. P0, postnatal day 0. Figure panel created in BioRender (K.J.W., 2025; https://BioRender.com/f498903). Mass spectrometry analysis of (**B**) serum and (**C**) retina testosterone and progesterone in wild-type females (purple), males (blue), females with OVX (pink), and males with OCX (orange), 6 weeks postsurgery. Statistics performed via one-way analysis of variance (ANOVA) with Tukey’s multiple comparisons test. *N* ≥ 8 mice per group. Error bars = SEM. *P < 0.05, ***P < 0.001, and ****P < 0.0001.

Our observed sex difference in visual function began at 2 months of age, after mice reach sexual maturity and after the onset of disease in this model system (approximately postnatal day 18) (*37*–*39*). To assess the effect of the steroidal sex hormones on retinal neurodegeneration, we surgically removed the ovaries [bilateral ovariectomy (OVX)] and testis [orchiectomy (OCX)] from RHO P23H female and male mice at sexual maturity (6 weeks of age; Fig. 2A). This gonadectomy approach results in removal of the primary sex hormone–producing organs but leaves the remainder of the hypothalamic-pituitary-gonadal axis intact. While some tissues can locally synthesize these hormones via cholesterol (*40*–*42*), whether this occurs within the retina is unknown. We used mass spectrometric analysis of mouse serum and neural retinal samples to assess hormone concentrations with and without surgical manipulation. At 12 weeks of age, 6 weeks postsurgery, serum exhibited lowered testosterone and progesterone after gonadectomy (Fig. 2B). The retina also displayed detectable and significant changes in hormone concentrations similar to those found in the serum samples (Fig. 2C).

### OVX ameliorates the accelerated visual decline in RP females

**Fig. 3.**
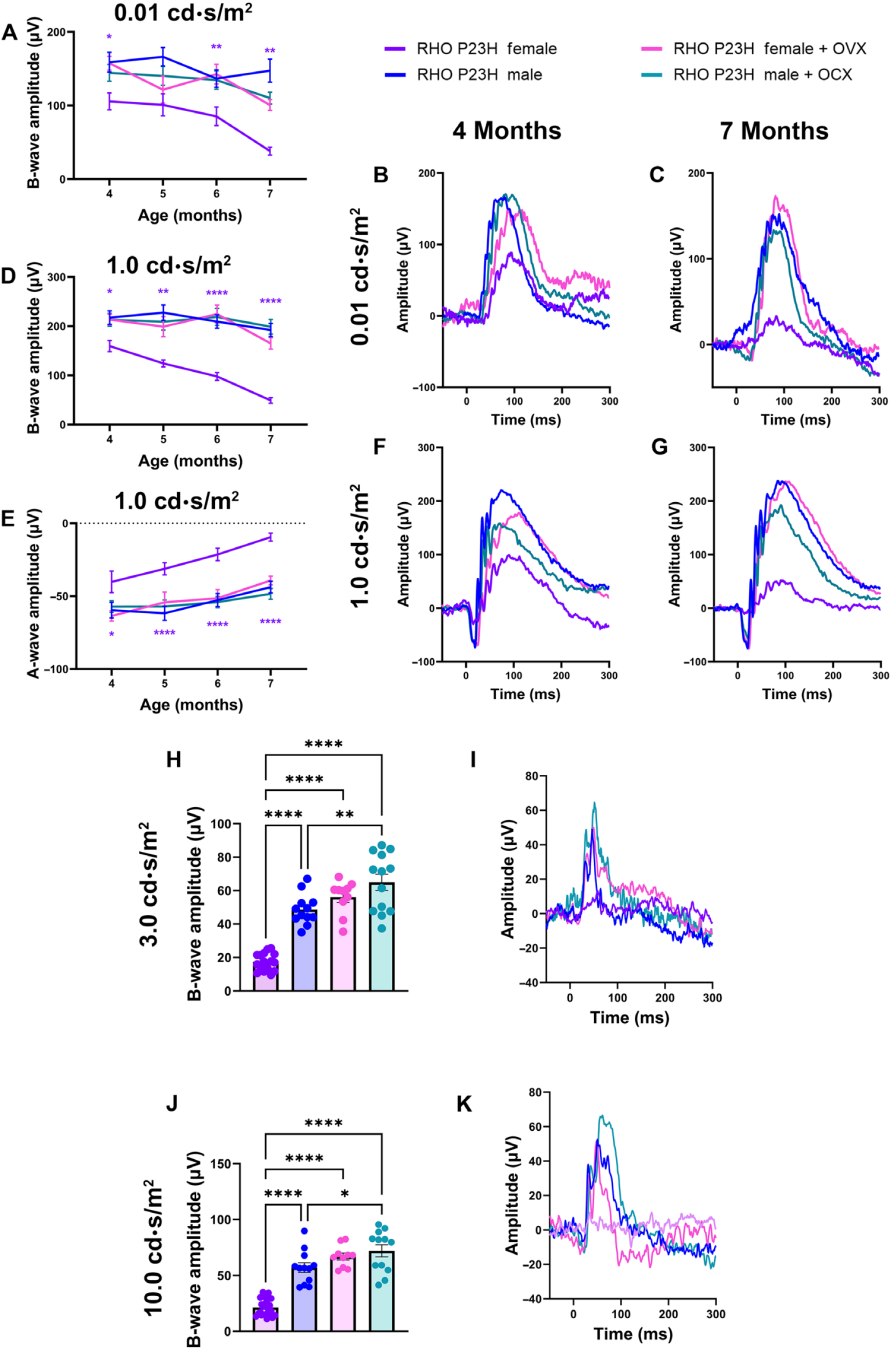
OVX ameliorates the accelerated female photoreceptor neurodegeneration. (**A**) Scotopic (0.01 cd·s/m^2^) b-wave amplitudes from 4 to 7 months of age in RHO P23H females (purple), males (blue), females with OVX (pink), and males with OCX (light blue). (**B** and **C**) Representative 0.01 cd·s/m^2^ scotopic traces. (**D**) Scotopic (1.0 cd·s/m^2^) b- and (**E**) a-wave amplitudes from 4 to 7 months of age and (**F** and **G**) representative traces. Statistics performed via two-way ANOVA with Tukey’s multiple comparisons test. Statistical results are shown for females compared to females + OVX and males compared to males + OCX. *N* ≥ 12 eyes. (**H**) Photopic amplitudes of 3.0 cd·s/m^2^ and (**J**) 10.0 cd·s/m^2^ at 7 months of age, (**I** and **K**) with respective representative traces. Statistics analyzed by one-way ANOVA with Tukey’s multiple comparisons test. *N* ≥ 10 eyes. Error bars = SEM. *P < 0.05, **P < 0.01, and ****P < 0.0001.

We assessed the effect of systemic hormone depletion on visual function for the RHO P23H mouse by ERG. Analysis of the rod photoreceptor function using the 0.01 cd⋅s/m^2^ scotopic setting revealed that there were no appreciable differences between intact males and males who received OCX. However, females that received OVX had a significant increase in visual function compared to intact females, which was not significantly different from males (Fig. 3, A to C). Scotopic ERG (1.0 cd·s/m^2^) showed similar results, where females with OVX had significantly higher ERG values for both the b- (Fig. 3, D, F, and G) and the a-wave amplitudes (Fig. 3, E to G), matching that of male littermates.

**Fig. 4.**
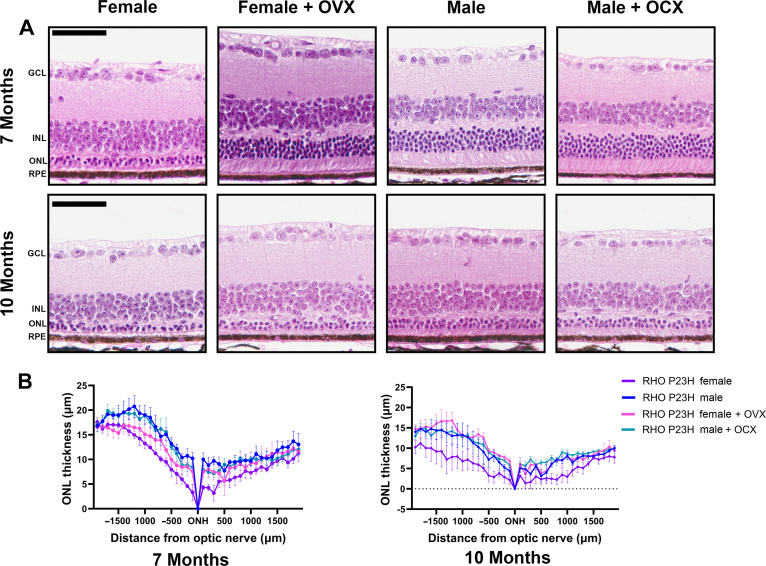
OVX ameliorates the loss of photoreceptor nuclei in females. (**A**) Hematoxylin and eosin–stained histology from 7- and 10-month-old RHO P23H female and male mice with and without OVX or OCX, respectively. Scale bars, 50 μm. (**B**) Quantification of ONL thickness at 100-μm distances spanning from the ONH at both 7 and 10 months of age. Statistics performed via two-way ANOVA with Tukey’s multiple comparisons test. Statistics for ONL quantification are available in data S1. *N* ≥ 3 mice per group with 38 measurements per retina. Error bars = SEM.

In RP, as the rod photoreceptor neurons die, the cone photoreceptors experience secondary death, leading to a loss of central vision in later stages of retinal degeneration. Therapeutic approaches center around preserving cone photoreceptor function, as this allows patients to keep their central visual field—required for recognizing faces, driving, etc.—as long as possible. Thus, we assessed photopic signals at late-stage retinal degeneration in the RHO P23H mice to investigate cone neuron–specific function. Both light-adapted 3.0 cd·s/m^2^ (Fig. 3, H and I) and 10.0 cd·s/m^2^ (Fig. 3, J and K) photopic settings revealed that female mice had a significantly reduced signal, while females with OVX retained a higher photopic signal similar to that of male photopic amplitudes. We did note that male mice with OCX had a significantly higher photopic reading compared to intact males at both light-intensity settings (Fig. 3, H to K). We next assessed the late structural changes in the retina and found that male mice who received OCX had no difference in ONL thickness compared to intact males. Female mice who received OVX had a thicker ONL than intact females at both 7 and 10 months of age, matching the photoreceptor survival found in male mice and congruent with the ERG results (Fig. 4, A and B).

### OVX affects degenerating but not healthy retinal neurons

To determine whether the steroidal sex hormones affect the retinal neurons outside of a disease context, we performed OVX on wild-type females and assessed visual function by ERG over time compared to intact males and females. We found no difference between female mice that had received OVX and intact females or males through 7 months of age for 0.01 cd·s/m^2^ scotopic and 1.0 cd·s/m^2^ scotopic b- and a-wave amplitudes (fig. S1, A to E). We also analyzed retinal histology from these mice at 7 months of age. No difference was observed for ONL thickness in any of the 38 regions analyzed throughout the retina (fig. S1, F and G).

### Exogenous estradiol affects male and female retinas without altering progesterone or testosterone levels

**Fig. 5.**
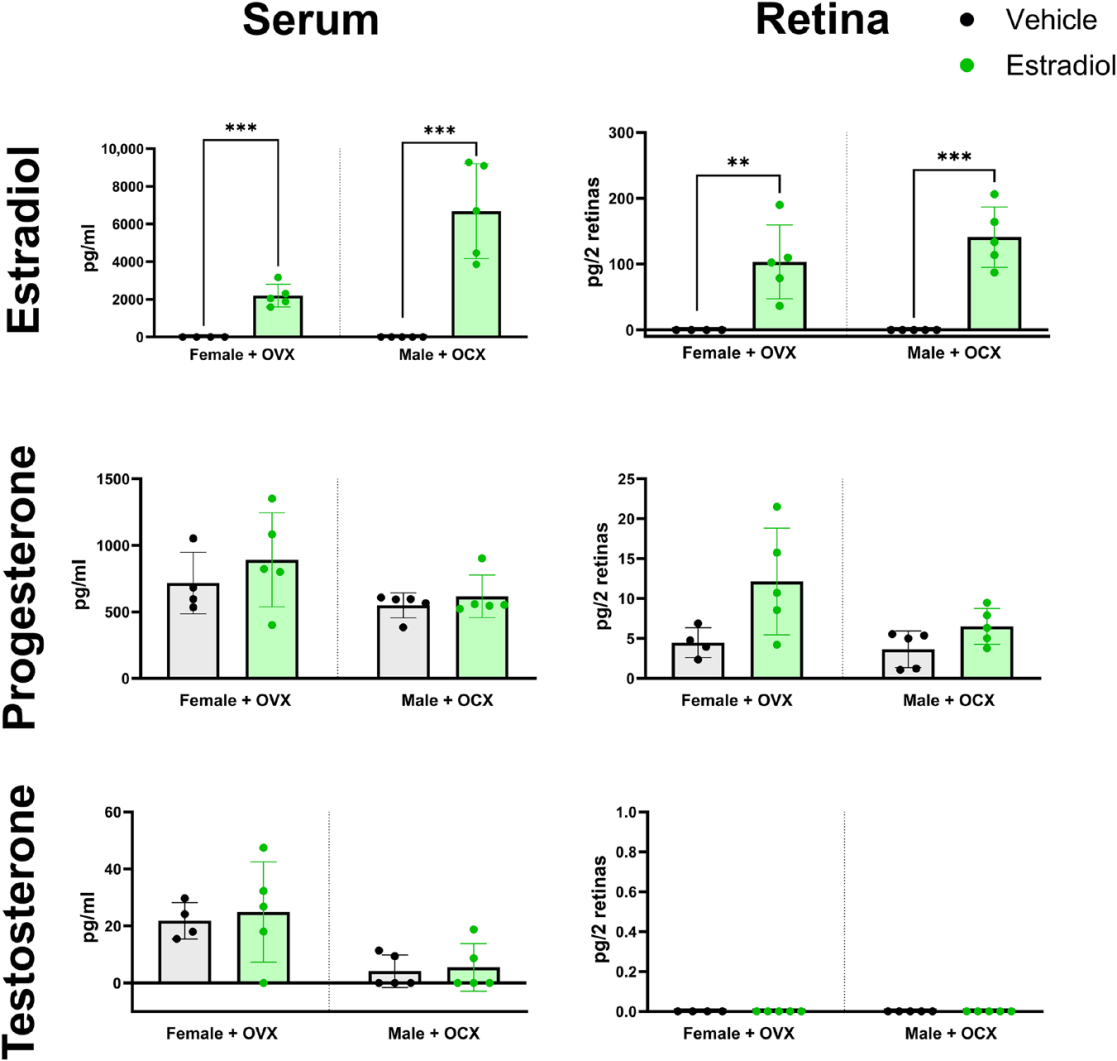
Exogenous 17β-estradiol affects male and female retinas without altering progesterone or testosterone levels. Mass spectrometry analysis of the serum and retina levels of estradiol, progesterone, and testosterone in 12-week-old wild-type mice that received either a vehicle injection (black) or an injection of estradiol (green). All mice received surgical hormone depletion (OVX and OCX) at 6 weeks of age. Statistics analyzed via *t* test (α = 0.05). Error bars = SD. **P < 0.01 and ***P < 0.001.

To determine whether systemic estradiol could directly affect photoreceptor health in RP mice, we first assessed whether a systemically administered hormone could cause measurable increases in estradiol levels in the serum and retina tissue. To test this, we delivered a subcutaneous injection of either estradiol or vehicle to 12-week-old wild-type mice that received hormone depletion surgery (OVX or OCX) at 6 weeks of age. We then collected serum and neural retina tissue 1 hour postinjection. Mass spectrometric analysis showed that both estradiol-injected females with OVX and males with OCX had a significant increase in serum estradiol levels (Fig. 5). Analysis of the neural retina tissue confirmed that retinas also had significantly higher estradiol levels. Delivery of exogenous estradiol via systemic injection did not result in any significant changes for either progesterone or testosterone in the serum or retina tissue. Testosterone levels were undetected for all retinal samples consistent with our earlier findings that OVX females and OCX males had no detectable testosterone in the neural retina (Fig. 2C).

### Reintroduction of estradiol at midstage of disease exacerbates visual function loss in females, but not males, with RP

**Fig. 6.**
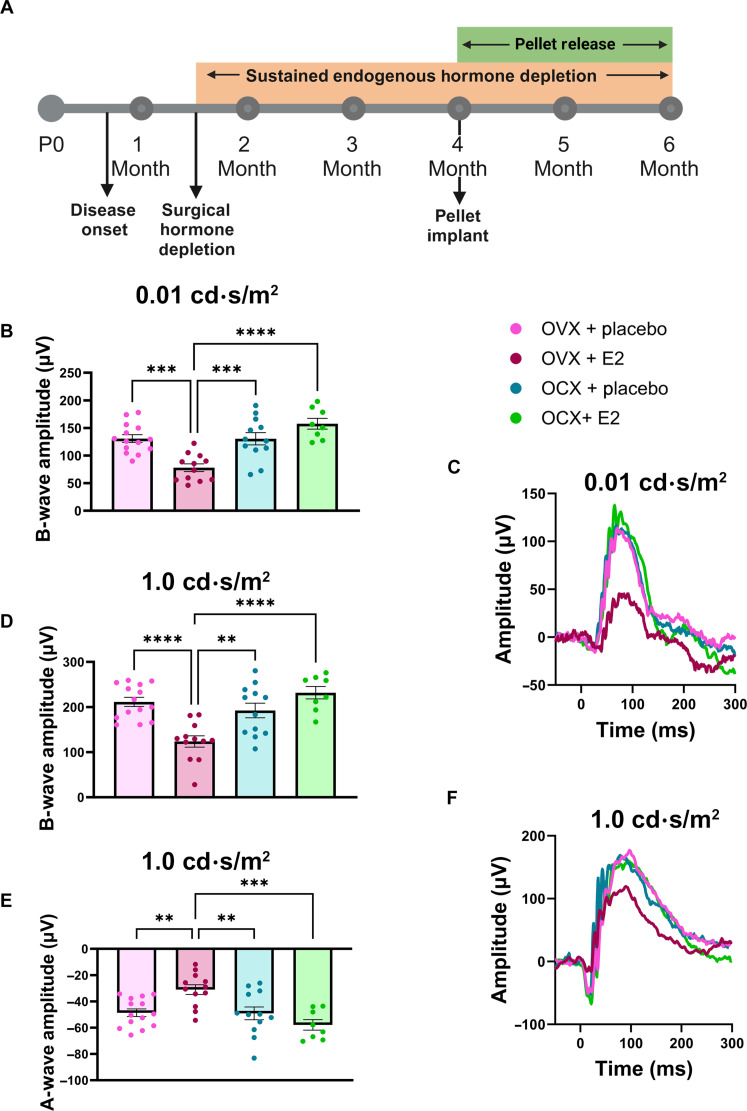
Reintroduction of estradiol at midstage of disease exacerbates visual function loss in females, but not males, with RP. (**A**) Timeline depicting surgical procedure at 6 weeks of age, after disease onset, followed by sustained hormone depletion through the duration of the study and reintroduction of estradiol via implanted pellet at 4 months of age. Figure panel created in BioRender (K.J.W., 2025; https://BioRender.com/k14c552). (**B**) ERG results from female RHO^P23H/+^ (RP) mice implanted with either a placebo (pink) or estradiol (E2) (burgundy) slow-release pellet and male RP mice implanted with either placebo (blue) or E2 (green) pellets. Scotopic ERG (0.01 cd·s/m^2^) b-wave amplitudes, (**D**) scotopic ERG (1.0 cd·s/m^2^) b-wave, and (**E**) a-wave amplitudes. Representative ERG traces for the (**C**) 0.01 cd·s/m^2^ and (**F**) 1.0 cd·s/m^2^ light intensity settings. Statistics analyzed via one-way ANOVA (α = 0.05) with Tukey’s multiple comparisons test. *N* ≥ 8 eyes per group. Error bars = SEM. **P < 0.01, ***P < 0.001, and ****P < 0.0001.

As OVX had a significant effect on alleviating the sex difference in RHO P23H females, we next sought to test whether reintroducing the female sex hormones could exacerbate vision loss. We performed gonadectomies in RHO P23H mice at 6 weeks of age, followed by surgically implanting slow-release, estradiol-containing pellets at 4 months of age (Fig. 6A). Two months after pellet implantation, visual function was assessed with scotopic ERG. Scotopic recordings (0.01 cd⋅s/m^2^), indicative of rod photoreceptor function, were significantly lower in OVX females implanted with estradiol-containing pellets compared to placebo-implanted OVX females and OCX males (Fig. 6B). Scotopic recordings (1.0 cd⋅s/m^2^), reflecting visual function from the photoreceptors through to the bipolar cells, followed a similar outcome, where estradiol-implanted OVX female mice had a significantly lower visual response in both the b- (Fig. 6D) and the a-wave amplitudes (Fig. 6E) compared to all groups tested. OCX males implanted with estradiol-containing pellets showed no difference compared to placebo-implanted OCX males at any of the light settings tested (Fig. 6, B, D, and E). Representative ERG traces for both 0.01 cd·s/m^2^ and 1.0 cd·s/m^2^ light intensity settings further illustrate that the OVX females with estradiol-implanted pellets displayed diminished visual function compared to other groups (Fig. 6, C and F).

### Addition of 17β-estradiol does not affect visual function for healthy male and female mice

As hormonal medications are considered safe for most of the population, we hypothesized that hormone delivery would only affect the RP females and not healthy female mice. To test this hypothesis, we implanted intact female and male wild-type mice at 3 months of age with either estradiol or placebo pellets and measured visual function 2 months postimplantation. We found that both female and male wild-type mice implanted with estradiol pellets had no changes in visual function at either the 0.01 cd·s/m^2^ or 1.0 cd·s/m^2^ light intensity setting (fig. S2, A, C, and D). Representative ERG traces also illustrated that estradiol-implanted wild-type females and males had no detectable changes in visual function compared to placebo-implanted mice (fig. S2, B and E).

### Transcriptomic analysis of the neural retina detects significant elevation in genes related to inflammatory and endoplasmic reticulum stress–induced apoptotic responses in RP females compared to RP males

**Fig. 7.**
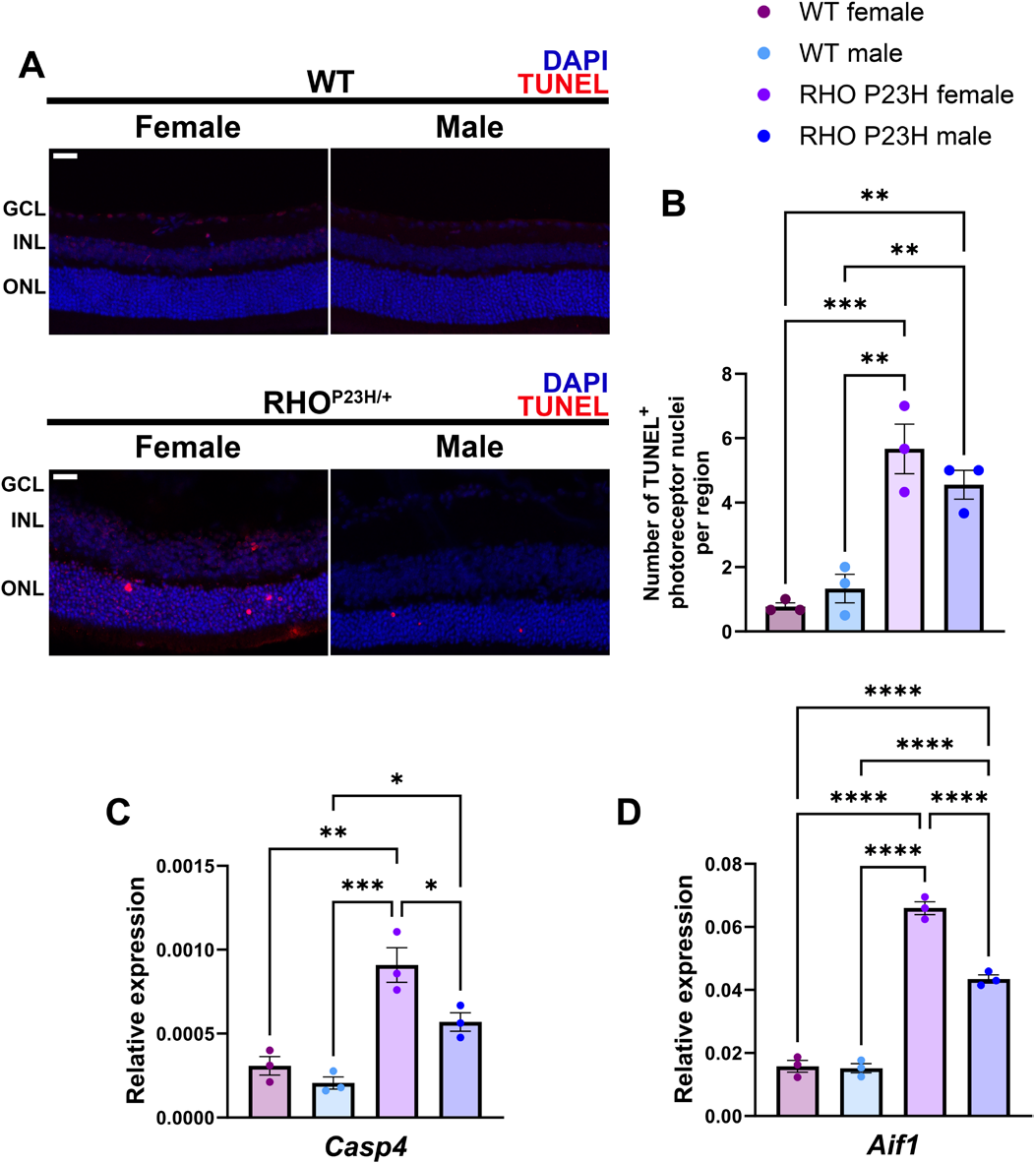
RP females display a significant elevation of genes related to pyroptosis, inflammatory, and endoplasmic reticulum stress–induced apoptotic responses compared to RP males. (**A**) TUNEL staining in 7-week-old RHO^P23H/+^ (RP) and wild-type (WT) retinas. DAPI (4′,6-diamidino-2-phenylindole) nuclear stain in blue and TUNEL in red. Scale bars, 25 μm. (**B**) Quantification of TUNEL-positive photoreceptor nuclei per region. *N* = 3 mice per group. Relative expression as measured by qPCR of the neural retina tissue for (**C**) *Casp4* (caspase 4) and (**D**) *Aif1* (allograft inflammatory factor 1). *N* = 3 retinas per group. WT females, burgundy; WT males, light blue; RP females, purple; RP males, blue. Statistics analyzed via one-way ANOVA (α = 0.05) with Tukey’s multiple comparisons test. Error bars = SEM. *P < 0.05, **P < 0.01, ***P < 0.001, and ****P < 0.0001.

Our observed accelerated visual decline in female RHO P23H mice indicates an underlying cellular stress or death pathway that is exacerbated in female mice concomitant with the presence of female steroidal sex hormones. Thus, we determined whether there was a sex difference in cell death using terminal deoxynucleotidyl transferase–mediated deoxyuridine triphosphate nick end labeling (TUNEL) staining on retinal sections from male and female RP mice and wild-type controls. The eyes were collected and sectioned from 7-week-old mice, when the mice have reached sexual maturity but have minimal changes in ONL thickness. The number of TUNEL-positive photoreceptors within a defined region of each retinal section was counted. We found that both female and male RHO P23H mice had a significant increase in TUNEL-positive photoreceptor nuclei compared to wild-type mice, consistent with prior literature (*43*). Female RHO P23H mice displayed a greater number of TUNEL-positive photoreceptor nuclei than their male counterparts; however, on the basis of variation between individual mice, this was not statistically significant (Fig. 7, A and B). Because TUNEL staining, indicative of DNA fragmentation, marks cells that are undergoing many forms of cell death, we further investigated different cell death pathways to identify those in which sex may be an important modifier. We performed a pan-cell death quantitative polymerase chain reaction (qPCR) array. Neural retina samples were collected from 12-week-old male and female wild-type and RHO P23H mice, and this array captured genes involved in intrinsic and extrinsic apoptosis, autophagy, necroptosis, pyroptosis, ferroptosis, NETosis, and parthanatos (figs. S3, A to D, and S4, A to D). We detected significant changes in multiple cell death pathways between RP and wild-type mice, indicative of active cell death mechanisms at play for this disease. There were two genes, *Casp4* and *Aif1*, that showed a significant difference between female and male RHO P23H mice, in addition to both being significantly altered compared to wild-type mice (Fig. 7, C and D).

## DISCUSSION

Investigation into mechanisms that have profound effects on underlying neurodegeneration in an incurable disease such as RP is of great clinical interest, as the field seeks to find ways to slow neuronal loss and preserve function. In this study, we investigated and defined the sexually dimorphic vision loss between male and female mice with a RHO P23H knock-in mutation that mimics the most prevalent form of autosomal dominant RP (*25*–*32*). We defined the contribution of sex-specific hormones on the manifestation of sex differences in RHO P23H RP, as hormonal interactions present opportunities for therapeutic intervention. Through hormone depletion studies in vivo, we found that depletion of the female sex hormones resulted in a near total amelioration of the functional and structural sex difference in our RP model of neurodegeneration, while delivery of estradiol worsened degeneration rates for female RP mice.

In studies of retinal degenerative disease in rodent models, beneficial effects of hormone therapy have been reported (*44*–*50*). For instance, one group found that topical administration of progesterone to the eye resulted in an alleviation of oxidative stress and reduction in apoptosis in the *rds* mouse model of RP (*44*). In this study, however, a sexually dimorphic phenotype was not reported, and the study was mainly conducted at postnatal day 21, before the onset of sexual maturity. Another study reported a neuroprotective effect of 17β-estradiol on the retinas of mice undergoing *N*-methyl-d-aspartate retinal toxicity, but they assessed 6- to 8-week-old male mice and females were not included (*47*). In this study, our data suggest that an interaction of hormone signaling with genetic mutations causal to neurodegenerative disease reduces function and neuronal cell survival in females. As depletion or addition of female sex hormones does not affect retinal neuron function or survival in a healthy retina, the hormones likely interact within the cell stress response or cell death mechanisms underlying the rhodopsin genetic mutation. Thus, understanding the precise mechanisms by which the female sex hormones affect this particular genetic form of RP can provide critical insight into which genetic mutations implicated in retinal degenerative disease (where more than 270 genes are associated with these disorders) (*7*), and other neurodegenerative disorders may present with a sexually dimorphic phenotype. It may also inform mechanisms at play in neurodegenerative conditions such as age-related macular degeneration and Alzheimer’s disease where sex differences have already been noted (*1*–*5*).

Hormonal medications are among the most widely prescribed medications in the US (*35*, *36*), and it would be expected that delivery of hormones to healthy individuals would not have an adverse effect on vision. In support of this, we found that injection of estradiol was able to elevate the hormone concentration in both the serum and the retina of wild-type mice, but that wild-type male and female mice had no changes to visual function. Furthermore, hormone depletion via OVX also caused no detectable changes in visual function or retinal cell survival in wild-type mice. These data indicate that although hormonal medications may be safe for most of the population, care needs to be taken in prescribing these medications for patients carrying mutations such as RHO P23H or with other neurodegenerative diseases.

Depletion of hormones by OCX, as well as injection of estradiol, in male RP mice did not cause a detectable change in visual function. As sex hormones carry out biological functions by binding to hormone receptors (*9*–*18*), it may be that the receptor levels within the retina are reduced in males compared to females. If this is the case, although estradiol is present, then there is a limit on receptor availability for signaling activity. It is also possible that the female hormones specifically affect pathways critical for photoreceptor health in female RP mice, but these pathways are not driving photoreceptor degeneration in male RP mice. Our cell death qPCR array indicated that similar cell death pathways are affected in both male and female RP mice but that females have a significantly higher activation of genes responsive to pyroptosis, inflammation, and endoplasmic reticulum stress–induced apoptosis compared to RP males (*51*–*58*). In support of these findings, studies have linked these cellular responses to the retina and retinal degenerative disease (*31*, *32*, *51*, *55*–*58*). For example, a study that examined transcriptomic datasets for both mouse and human retina samples also found an increase in inflammation for females with RP compared to males (*23*). They noted this effect in the *rd10* mouse model of RP, a mouse carrying a different genetic mutation than the model in this study, suggesting common pathways affected by sex in these two genetic causes of RP. An additional study found elevated inflammatory responses in females compared to males in relation to aging of the retinal pigment epithelium, concomitant with losses in ERG responses (*59*). Future investigation is needed to explore the precise mechanisms within the retina by which the sex hormones alter the rate of disease and affect the health and survival of the photoreceptor neurons. These investigations will need to take into account cellular stress and death pathways during the long-term progression of these neurodegenerative diseases, considering acute versus chronic responses, as well as hormone manipulation. In addition, understanding how sex and sex hormones alter pyroptosis, inflammatory, and endoplasmic reticulum stress responses can open insight into mechanisms underlying not only photoreceptor degeneration in RP disease but also how sex alters these cellular responses in various tissues of the body.

Most human clinical studies investigate patients carrying several forms of neurodegenerative disease, such as RP, grouped together in longitudinal analysis, but individual mutation analysis is not typically considered. In animal studies for neurodegenerative diseases such as RP, groups typically examine the sexes separately (*60*), note that sex differences were not expected and therefore were not considered (*28*), use homozygous mutant models where degeneration occurs before the onset of sexual maturity (*26*, *29*), and/or perform experiments early during degeneration before a large variation between male and female mice would be easily noticed (*28*, *30*). However, sex differences have been noted for mouse models of retinal degenerative disease (*21*, *22*, *60*), albeit with no link to the mechanism. As other retinal degenerative diseases such as age-related macular degeneration and diabetic retinopathy have also noted sex differences (*2*, *61*, *62*), our data argue that biological sex as a variable should be rigorously tested in preclinical models to avoid biasing of resultant data.

**Fig. 8.**
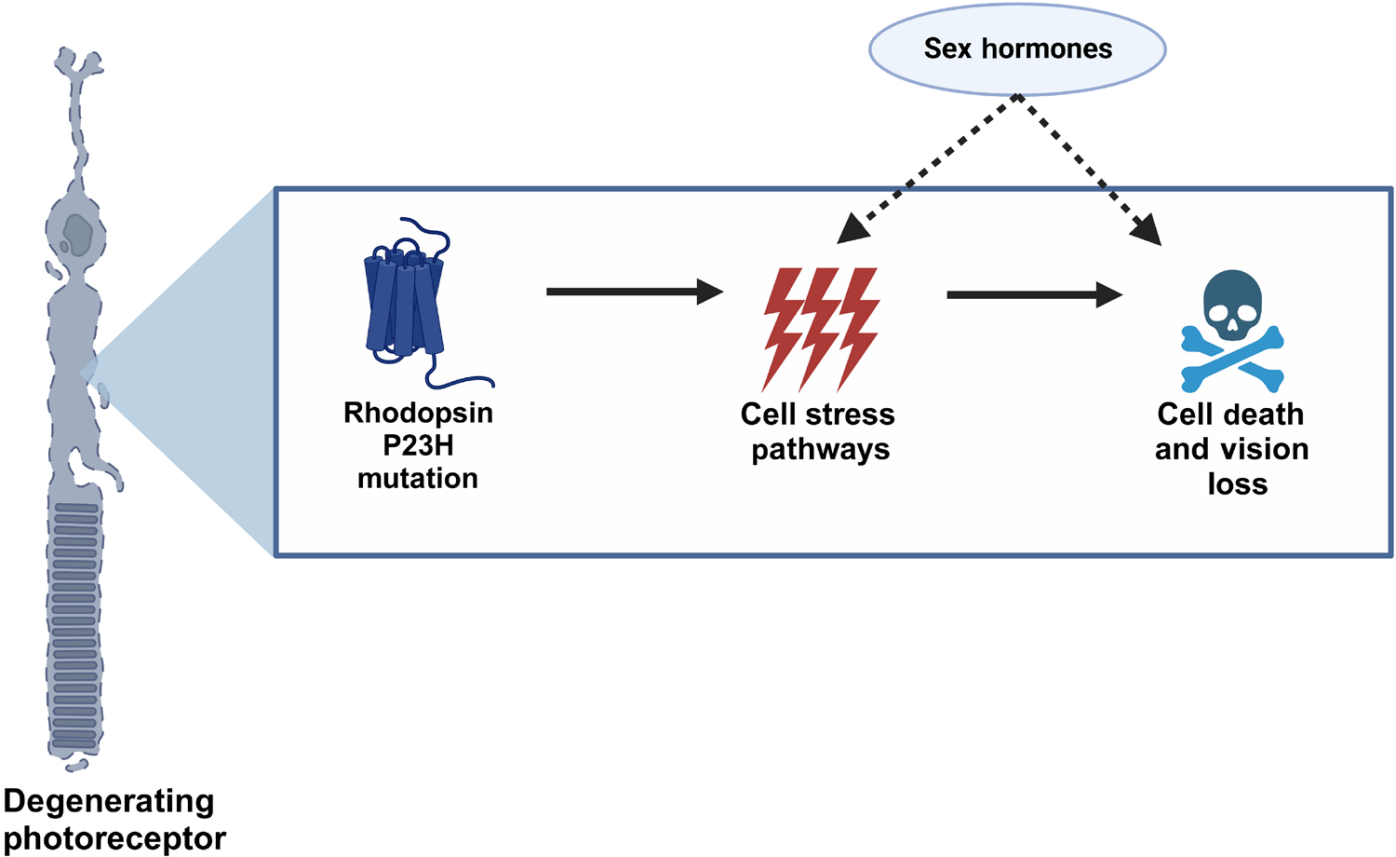
Sex hormones affect cellular pathways underlying the RHO P23H mutation in photoreceptor neurons. Graphical schematic depicting our findings that female systemic sex hormones interact within pathways found in diseased, but not healthy, retinal neurons to exacerbate neurodegeneration in a sexually dimorphic manner. Figure created in BioRender (K.J.W., 2025; https://BioRender.com/m93w003).

Overall, our data highlight a systemic hormone-dependent interaction of the female sex hormones with the pathology and progression of an inherited retinal dystrophy that was previously thought to be unaffected by biological sex (Fig. 8). These important findings highlight the need to advocate for sex stratification in preclinical research, as well as natural history and clinical trial studies for patients with RP and other neurodegenerative diseases. It also marks the importance of record keeping of hormonal medications for these patients.

## MATERIALS AND METHODS

### Mouse lines and husbandry

All experiments were performed in accordance with the Association for Research in Vision and Ophthalmology Statement for the Use of Animals in Ophthalmic and Visual Research and were approved by the Animal Care and Use Committee at UT Southwestern Medical Center (UTSW; Animal Protocol Number, #2019-102840). RHO P23H mice were obtained from the Jackson Laboratory (RRID: IMSR_JAX:017628). RHO P23H homozygous mice were bred with C57BL/6J mice (the Jackson Laboratory, Bar Harbor, ME, RRID: MSR_JAX:000664) to obtain the mice used in this study. C57BL/6J mice were used as wild-type controls. In addition, heterozygous RHO P23H breeding was used, and littermates were analyzed for sexual dimorphism in disease, which occurred only in mice carrying the RHO P23H mutation. All mice were maintained in approved animal facilities at UTSW and were kept on a normal light-dark cycle (12/12 hours). Food and water were available ad libitum throughout the experiment. Experiments were conducted monthly, with a month being measured as a cycle of 28 days. Animals of both sexes were used in this study, and data were sex-stratified.

### Electroretinography

Mice were dark-adapted for at least 12 hours, manipulations were conducted under dim red-light illumination, and recordings were made using the Celeris ERG system by Diagnosys LLC (Lowell, MA, USA) following the previous methods (*63*, *64*). Briefly, the pupils were dilated using topical 2.5% phenylephrine hydrochloride (Akorn Inc.) and 1% tropicamide (Sandoz). Mice were anesthetized by intraperitoneal injection of 0.1 ml/10 g body weight of anesthesia [1 ml of ketamine (100 mg/ml) and 0.1 ml of xylazine (20 mg/ml) in 8.9 ml of 1× phosphate-buffered saline (PBS)]. Body temperature was maintained at 37°C during the testing. The mouse eyes were covered in a 0.3% hypromellose gel (GenTeal, Alcon). Scotopic retinal responses were recorded at 0.01 and 1.0 cd·s/m^2^ white light intensity settings. A minimum of eight measurements were recorded and averaged for each light setting. Photopic ERG was recorded at 7 months of age, immediately following the scotopic ERG using the Celeris system. The mouse eyes were exposed to 10 min of white light, followed by flashes at two white light settings: 3.0 and 10.0 cd·s/m^2^, each one averaging at least 15 sweeps.

### Gonadectomies

OVX and OCX were performed as previously described (*65*). Briefly, mice were shaved, and subcutaneous pain medications of meloxicam (5 mg/kg) and buprenorphine sustained-release (SR) (1.0 mg/kg) were delivered. Anesthesia was maintained using 2% isoflurane via a precision vaporizer. The eye surfaces were protected using artificial tear ointment, and the body temperature was maintained at 37°C throughout the procedure. OCX was performed via a single midline incision in the ventral scrotal sac where both testes were removed. OVX was performed via two dorsal-lateral incisions into the abdominal wall where the ovaries were isolated and severed by the distal uterine horn, resulting in the removal of the oviduct and ovary. In both cases, internal layers were closed using 4-0 absorbable suture material, and skin wounds were closed with wound clips. Confirmation of OVX success was monitored via estrus cycle cessation observed via vaginal cytology and visual identification of estrus phases (*66*).

### Retinal histology and quantification

Mice were euthanized following the institutional guidelines. Enucleation was performed by proptosing the eye and placing a curved pair of forceps behind the eye, near the optic nerve, and gently pulling outward, releasing the eye and a portion of the optic nerve as previously described (*63*, *64*). For paraffin-embedded sections, the eyes were fixed at room temperature and embedded in paraffin, sectioned, and stained with hematoxylin and eosin by Excalibur Pathology Inc. Each section contained the upper and lower retina as well as the posterior pole. Retinal sections were imaged using light microscopy (Hamamatsu NanoZoomer S60, UTSW Whole Brain Microscopy Facility, RRID: SCR_017949). Measurement of ONL thickness was done using the Hamamatsu NDP software to visualize Nanozoomer scanned slides. The thickness of the ONL in micrometers was measured every 100 μm on either side of the optic nerve head. For cryosections, the eyes were enucleated, and a hole in the cornea was made using a 23-gauge needle before fixing the eye in 4% paraformaldehyde for 45 min at room temperature. The eyes were transferred to a sucrose gradient of 10, 20, and 30% sucrose each for an hour, followed by an additional 30% sucrose incubation at 4°C overnight. The next day, the eyes were placed in optimal cutting temperature (OCT) media (Tissue-Tek) and frozen at −80°C. Sectioning was performed on a cryostat (Leica Biosystems) at a thickness of 12 μm. Frozen sections were collected on superfrost plus slides and stored at −20°C until ready for use.

### Retina dissection and serum collection

Before mass spectrometry analysis, blood was collected by submandibular bleeding using a 5-mm lancet. Whole blood was collected into a 1.5-ml uncoated centrifuge tube and allowed to coagulate at room temperature for 30 min to an hour. Next, the sample was spun in a 4°C centrifuge for 10 min at ×3000*g*. The serum supernatant was pipetted off the top and frozen at −80°C until ready for processing. After blood collection, the mice were euthanized via cervical dislocation before dissection of the retinas. A razor blade was used to slice a midline incision into the cornea. Next, a curved pair of forceps was used to remove the lens, followed by the removal of the neural retina, which was placed in power bead tubes (QIAGEN) and flash frozen in liquid nitrogen. The retinas were stored at −80°C until ready for processing.

### Mass spectrometry

Retinas: Retinas were placed in bead beater tubes (PowerBead Tubes, Ceramic 2.8 mm, QIAGEN, Germany) with 1 ml of 30% methanol and 20 μl of stable isotope–labeled steroid cocktail and homogenized using 5.50-ms^−1^, 10-s speed time (Bead Ruptor 24, Omni, Kennesaw, GA). The samples were vortexed for 30 s and then let stand for 5 min. The samples were centrifuged at ×4415*g* for 5 min. The steroids were isolated from the sample using solid-phase extraction [SPE; Evolute Express ABN (30 mg/1 ml), Biotage, Charlotte, NC]. Columns were sequentially conditioned with 1 ml of acetonitrile, 1 ml of methanol, and 0.5 ml of water. All SPE work was carried out with a positive pressure manifold (Biotage, Charlotte, NC) operated at 6 psi (41.37 kPa). The samples were loaded onto the conditioned columns and washed with 0.5 ml of water and then 0.5 ml of 30% methanol, both of which were discarded. Steroids were eluted with two 0.5 ml of aliquots of acetonitrile into a 2-ml polypropylene plate (2-ml Square 96-Well Plate with 100 μl of Tapered Reservoir; Analytical Sales, Flanders, NJ). The samples were evaporated under a gentle stream of nitrogen at 40°C and reconstituted in 200 μl of 0.2% formic acid and 50% methanol.

Serum: A 100 μl of aliquot of serum was added to a 2 -ml microcentrifuge tube along with 300 μl of water and 20 μl of stable isotope–labeled steroid cocktail. The mixture was vortexed for 5 s, then 200 μl of ZnSO_4_ was added, and it was vortexed again for 10 s. A 400 μl of aliquot of cold methanol was added, and the samples were vortexed for 30 s, then let stand for 5 min, and then centrifuged at ×4415*g* for 5 min. Steroids were isolated using the same SPE procedure described above for the retina samples. Samples were reconstituted with 200 μl of 0.2% formic acid and 50% methanol. Steroids were quantitatively measured using high-performance liquid chromatography–mass spectrometry as previously described (*67*, *68*).

### Subcutaneous hormone pellets

Slow-release pellets were obtained from the Innovative Research of America (Sarasota, FL) containing either a placebo (catalog no. NC-111) or 1.5 mg of 17β-estradiol (NE-121). Pellets were composed of a 90-day slow-release formulation, where the 1.5 mg of estradiol will release steadily over a 90-day window. Pellet dosage was based on previously published work (*69*–*71*). For pellet implantation, mice were shaved on the right lateral side of the neck, and subcutaneous pain medications of meloxicam (5 mg/kg) and buprenorphine SR (1.0 mg/kg) were delivered. Anesthesia was maintained using 2% isoflurane via a precision vaporizer. Eye surfaces were protected using artificial tear ointment, and body temperature was maintained at 37°C throughout the procedure. The skin on the lateral side of the neck was prepared following the aseptic techniques before a small incision into the skin was made where the pellet was inserted between the skin and the muscle layers, followed by closure of the skin using wound clips.

### Estradiol injections

Mice at 12 weeks of age were given a single subcutaneous injection of β-estradiol (0.1 μg/μl; Sigma-Aldrich, E2758) delivered in 50% dimethyl sulfoxide (DMSO)/50% sesame oil (Sigma-Aldrich, S3547), for a total delivery of 5 μg of β-estradiol per animal. Vehicle-injected mice received 50 μl of 50% DMSO/50% sesame oil subcutaneously. Retinas and blood were collected from mice 1 hour after injection.

### TUNEL assay

Cryosections of retinas were warmed to room temperature, and excess OCT medium was removed with three 1× PBS washes before performing the Click-iT Plus TUNEL Assay Kit (Thermo Fisher Scientific, catalog no. C10618). The assay was performed per the manufacturer’s protocol with no modifications. After TUNEL staining was complete, slides were counterstained with Hoescht (Thermo Fisher Scientific, reference no. 62249) for 5 min, followed by three 1× PBS washes. Slides were mounted with ProLong Gold Antifade Mountant (Thermo Fisher Scientific, catalog no. P36930). Slides were imaged on a Leica SP8 laser scanning confocal microscope using a 25× water immersion objective lens. Images were generated from a maximum-intensity projection of a z-stacked image taken of the retina. TUNEL-positive photoreceptor nuclei were counted in a 465-μm-wide retinal cross section located halfway between the optic nerve head and the ciliary body.

### Real-time qPCR

Retina samples were collected immediately following euthanasia and snap-frozen in liquid nitrogen, followed by storage at −80°C. RNA was isolated using the Qiagen RNeasy system. An on-column deoxyribonuclease digestion step was performed per the manufacturer’s protocols. Reverse transcription was performed using the High-Capacity RNA-to-cDNA Kit (Thermo Fisher Scientific, catalog no. 4388950). qPCR was performed using a QuantStudio 6 Pro Real-Time PCR system (Applied Biosystems) using a 10-μl reaction volume of PowerTrack SYBR Master Mix (Thermo Fisher Scientific, catalog no. A46109). Experimental primers were obtained from RealTimePrimers (Mouse Pan Cell Death Primer Library, item no. MPCD-I). Each biological replicate represents the average of three technical replicates normalized to the *U36b4* reference gene. The following primers were used: *U36b4* (forward) 5′-cgtcctcgttggagtgaca-3′ and (reverse) 5′-cggtgcgtcagggattg-3′. Data are shown as relative expression or 2^–ΔCt^.

### Statistical analysis

Data are reported as means ± SEM unless otherwise noted. GraphPad Prism Software (version 10.0) was used to generate graphs and perform statistical analysis. Data comparing three or more groups were analyzed using one-way analysis of variance (ANOVA), followed by Tukey’s multiple comparisons test. Data comparing two groups over multiple time points were analyzed using multiple two-tailed *t* tests with the Holm-Šidák’s method to correct for multiple comparisons. Data with two independent variables were analyzed using two-way ANOVA, followed by Tukey’s multiple comparisons test. For all applicable statistical tests, α was set to 0.05. Normal distribution and homogeneity of variance were determined by graphical analysis. See individual methods sections, results, and figure legends for specific testing methods. No mice were excluded from the analysis. A *P* value of less than 0.05 was considered significant, and measurements were performed blinded to experimental groups. All statistical analysis and *P* values are provided in data S1.
