# Supplementary material for: Female sex hormones exacerbate retinal neurodegeneration
Source: Sci Adv. 2025 Apr 11;11(15):eadr6211. doi: 10.1126/sciadv.adr6211 (PMC11988432; doi:10.1126/sciadv.adr6211)
Supplement: Supplementary file 1 — Figs. S1 to S4 Legend for data S1 [file sciadv.adr6211_sm.pdf]

Science Advances
AAAS

Supplementary Materials for

Female sex hormones exacerbate retinal neurodegeneration

Ashley A. Rowe *et al.*

Corresponding author: Katherine J. Wert, Katherine.Wert@UTSouthwestern.edu

Sci. Adv. **11**, eadr6211 (2025)
DOI: 10.1126/sciadv.adr6211

The PDF file includes:

Figs. S1 to S4
Legend for data S1

Other Supplementary Material for this manuscript includes the following:

Data S1

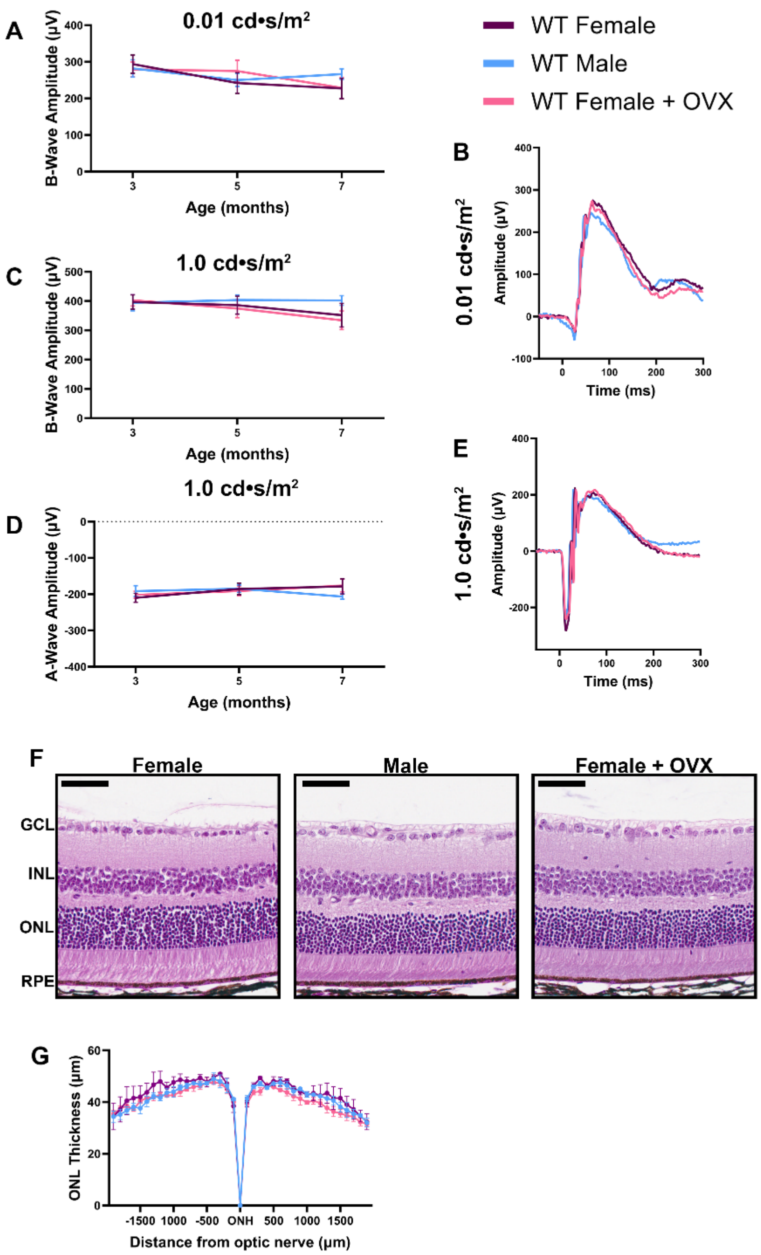

Figure S1. Steroidal sex hormone depletion does not affect healthy retinal neurons. (A) 0.01 cd•s/m^2 scotopic electroretinography (ERG) b-wave amplitudes, **(C)** 1.0 cd•s/m^2 scotopic ERG b- and **(D)** a-wave amplitudes from male (blue), female (purple), and females with bilateral ovariectomy (OVX; pink) in wild-type (WT) mice at three, five and seven months of age. Representative ERG traces for the **(B)** 0.01 cd•s/m^2 and **(E)** 1.0 cd•s/m^2 scotopic ERG settings for all three groups at seven months of age. Statistics analyzed via Two-way ANOVA (α=0.05) with Tukey's multiple comparisons test. N = 10 eyes per group. **(F)** H&E-stained histology of retinas from seven-month-old WT females, males, and females + OVX. **(G)** Quantification of the outer nuclear layer (ONL) thickness spanning either side of the optic nerve head (ONH) for all groups. GCL, ganglion cell layer; INL, inner nuclear layer; RPE, retinal pigmented epithelium. Statistics for ONL quantification available in supplemental material. Scale bar = 50 µm. N = 3 eyes per group. Error bars = SEM.

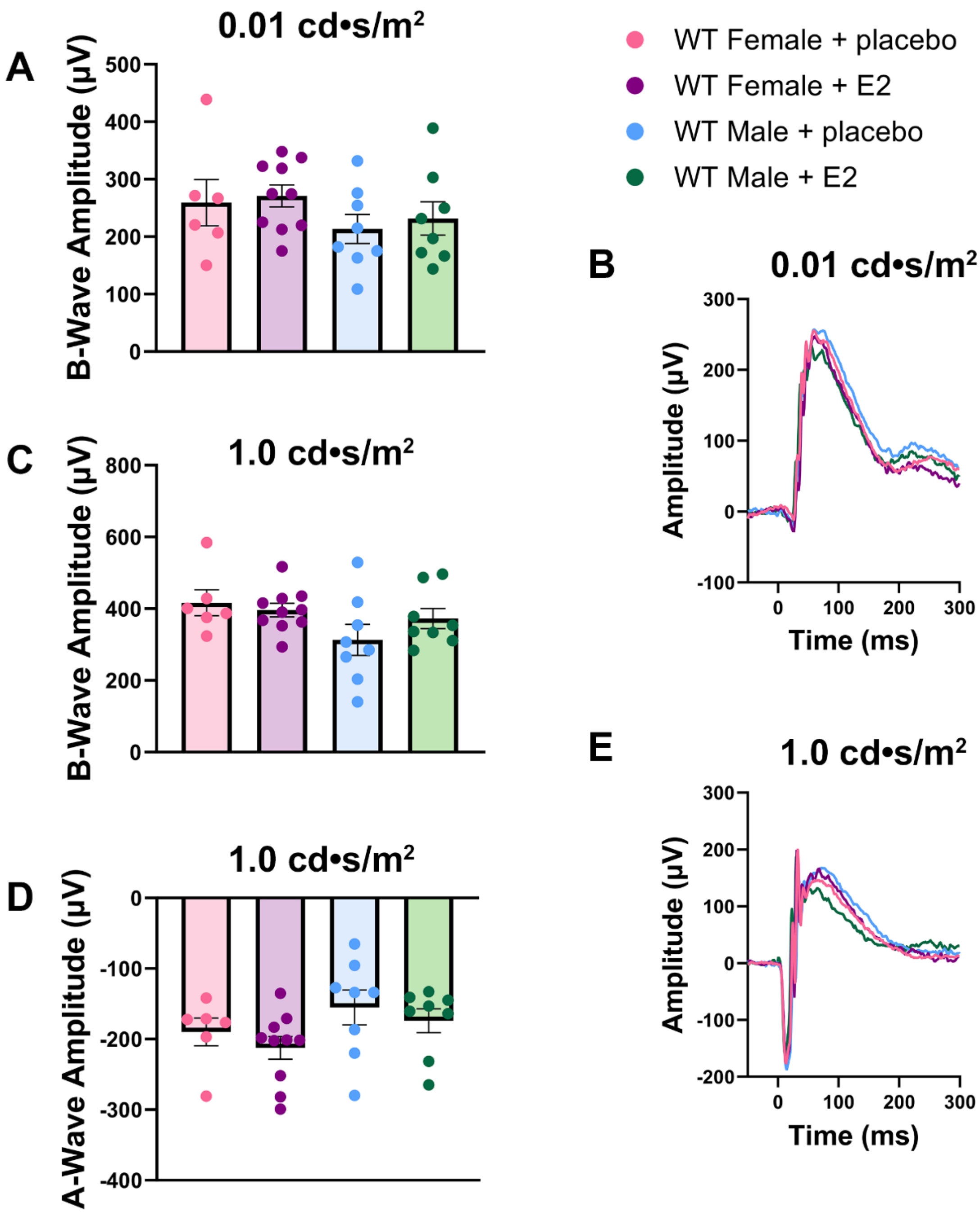

Figure S2. Addition of estradiol does not affect visual function for healthy male and female mice. Electroretinography (ERG) results from female wild-type (WT) mice implanted with either a placebo (pink) or estradiol (E2; burgundy) slow-release pellets and male WT mice implanted with either placebo (blue) or E2 (green) pellets. **(A)** 0.01 cd•s/m^2 scotopic ERG b-wave amplitudes, **(C)** 1.0 cd•s/m^2 scotopic ERG b-wave and **(D)** a-wave amplitudes. Representative ERG traces for the **(B)** 0.01 cd•s/m^2 and **(E)** 1.0 cd•s/m^2 scotopic ERGs. Mice were implanted at 3 months of age and imaged 2 months later. Statistics analyzed via One-way ANOVA (α=0.05) with Tukey's multiple comparisons test. N $\geq$ 6 eyes per group. Error bars = SEM.

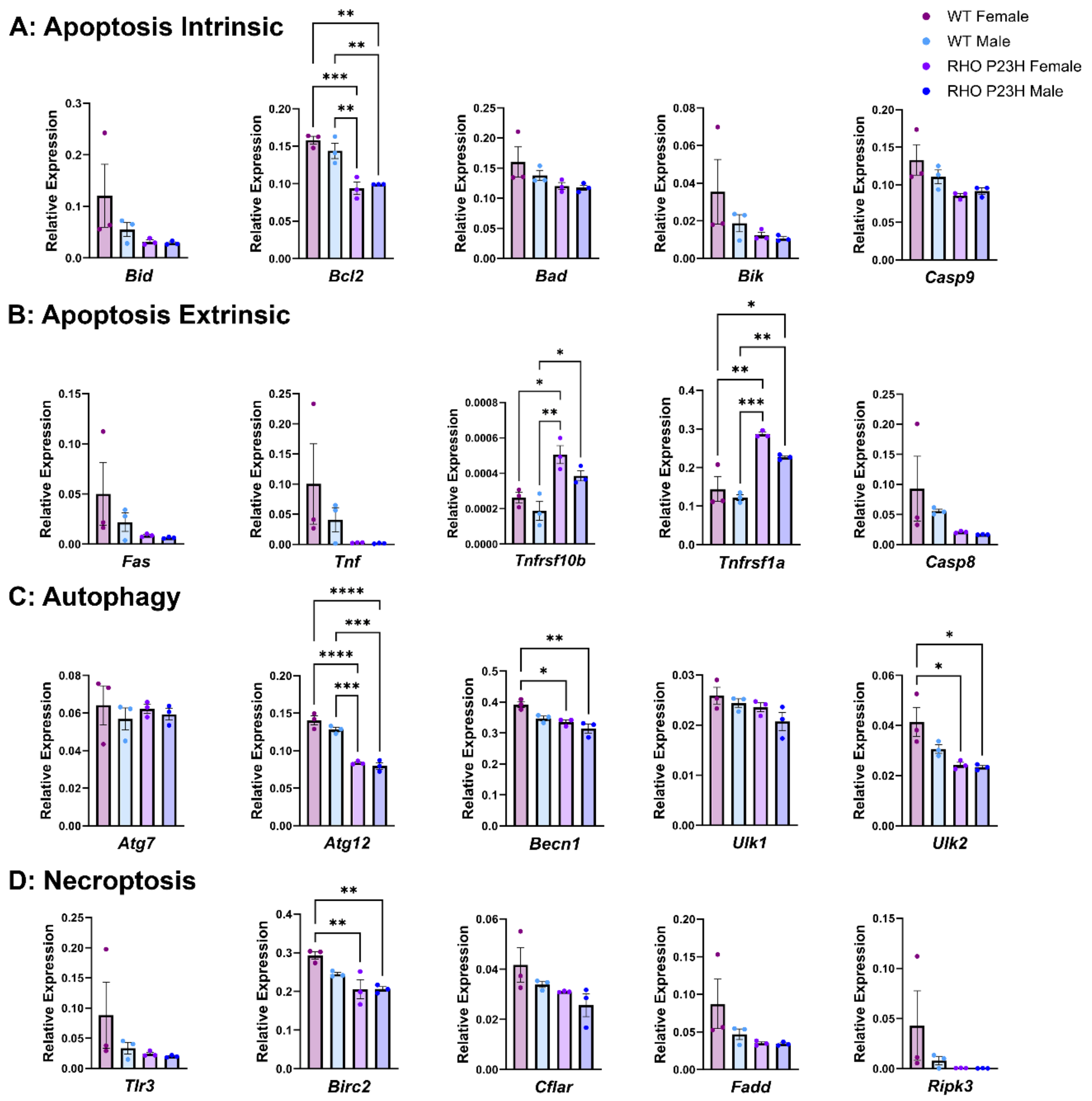

Figure S3. Changes in apoptosis, autophagy, and necroptosis in mice with retinitis pigmentosa (RP). Relative expression of transcripts as measured by qPCR for **(A)** intrinsic apoptosis, **(B)** extrinsic apoptosis, **(C)** autophagy, and **(D)** necroptosis between 3 month wild-type (WT) female (burgundy), WT male (light blue), RHO P23H female (purple), and RHO P23H male (dark blue) neural retina tissue. *Bid*, BH3 interacting domain death agonist. *Bcl2*, B-cell CLL/lymphoma 2. *Bad*, BCL-2-associated agonist of death. *Bik*, BCL-2 interacting killer. *Casp9*, Caspase 9. *Fas*, Fas cell surface death receptor. *Tnf*, Tumor necrosis factor. *Tnfrs10b*, TNF receptor superfamily 10b. *Tnfrsf1a*, TNF receptor superfamily 1a. *Casp8*, Caspase 8. *Atg7*, Autophagy related 7. *Atg12*, Autophagy related 12. *Becn1*, Beclin 1. *Ulk1*, unc-51 like autophagy activating kinase 1. *Ulk2*, unc-51 like autophagy activating kinase 2. *Tlr3*, Toll like receptor 3. *Birc2*, Baculoviral IAP repeat containing 2. *Cflar*, Casp8 and FADD-like apoptosis regulator. *Fadd*, Fas associated via death domain. *Ripk3*, receptor interacting serine-threonine kinase 3. Statistics analyzed via One-way ANOVA (α=0.05) with Tukey's multiple comparison's test. N= 3 retinas per group. Error bars = SEM. *, $p < 0.05$. **, $p<0.01$. ***, $p<0.001$. ****, $p<0.0001$.

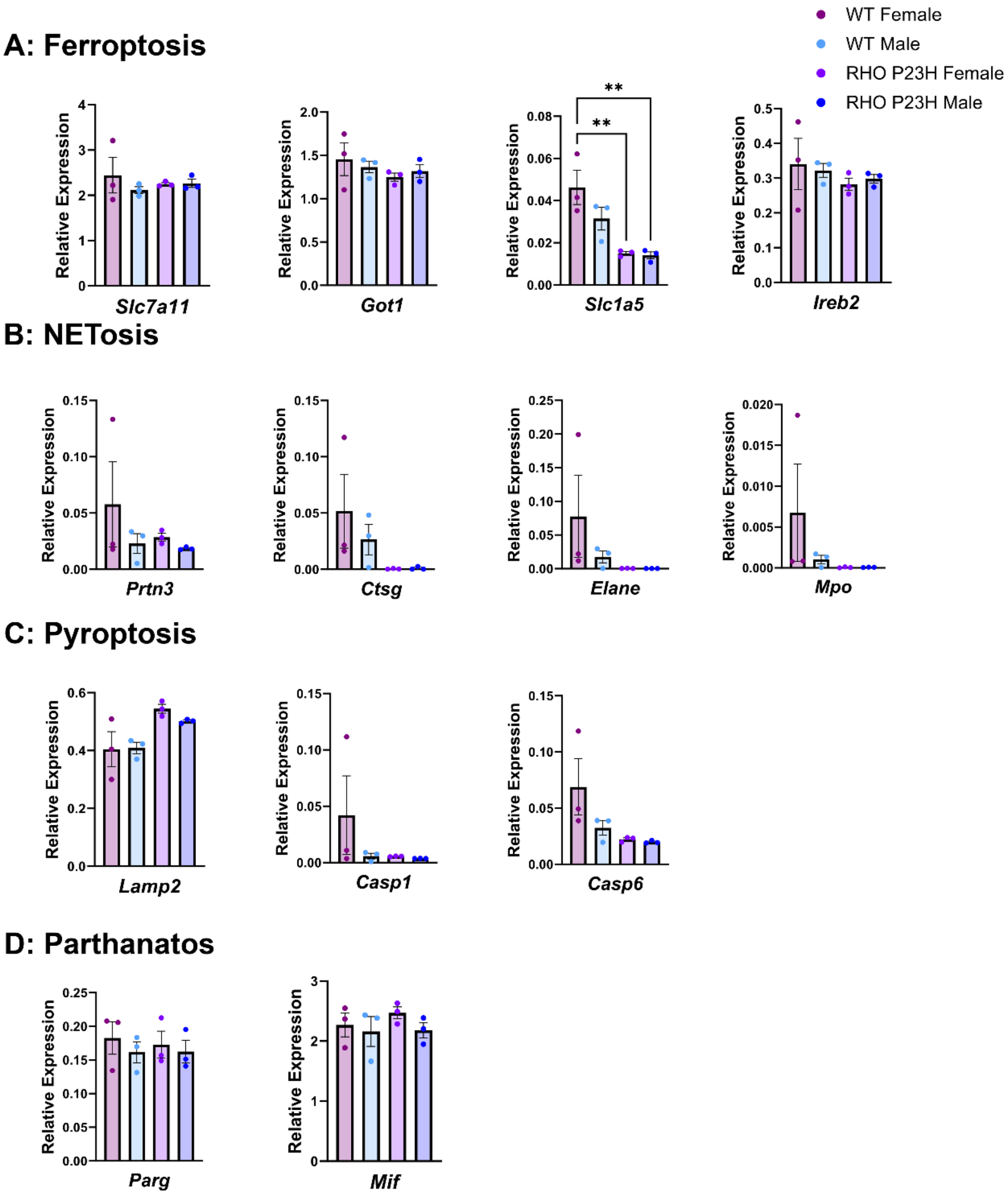

Figure S4. Changes in ferroptosis, NETosis, pyroptosis, and parthanatos in in mice with retinitis pigmentosa (RP). Relative expression of transcripts as measured by qPCR for **(A)** ferroptosis, **(B)** NETosis, **(C)** pyroptosis, and **(D)** parthanatos between 3 month wild-type (WT) female (burgundy), WT male (light blue), RHO P23H female (purple), and RHO P23H male (dark blue) neural retina tissue. *Slc7a11*, solute carrier family 7 member 11. *Got1*, glutamic oxaloacetic transaminase 1. *Slc1a5*, solute carrier family 1 member 5. *Ireb2*, iron responsive binding element protein 2. *Prtn3*, Proteinase 3. *Ctsg*, cathepsin G. *Elane*, elastase. *Mpo*, myeloperoxidase. *Lamp2*, lysosomal membrane-associated protein 2. *Casp1*, Caspase 1. *Casp6*, Caspase 6. *Parg*, poly (ADP-ribose) glycohydrolase. *Mif*, macrophage migration inhibitory factor. Statistics analyzed via One-way ANOVA (α=0.05) with Tukey's multiple comparisons test. N= 3 retinas per group. Error bars = SEM. **, p<0.01.

Data S1. Data values and statistical results. All data values and statistical results for each figure panel in a tabular format in Excel.
